# Amyloid-*β*_(25-35)_ Modulates the Expression of GirK and KCNQ Channel Genes in the Hippocampus

**DOI:** 10.1371/journal.pone.0134385

**Published:** 2015-07-28

**Authors:** Jennifer Mayordomo-Cava, Javier Yajeya, Juan D. Navarro-López, Lydia Jiménez-Díaz

**Affiliations:** 1 University of Castilla-La Mancha, Neurophysiology & Behavior Lab, CRIB, School of Medicine of Ciudad Real, Ciudad Real, Spain; 2 University of Salamanca, Department of Physiology & Pharmacology, Salamanca, Spain; University of Exeter, UNITED KINGDOM

## Abstract

During early stages of Alzheimer’s disease (AD), synaptic dysfunction induced by toxic amyloid-*β* (A*β*) is present before the accumulation of histopathological hallmarks of the disease. This scenario produces impaired functioning of neuronal networks, altered patterns of synchronous activity and severe functional deficits mainly due to hyperexcitability of hippocampal networks. The molecular mechanisms underlying these alterations remain unclear but functional evidence, shown by our laboratory and others, points to the involvement of receptors/channels which modulate neuronal excitability, playing a pivotal role in early Aβ-induced AD pathogenesis. In particular, two potassium channels that control neuronal excitability, G protein-coupled activated inwardly-rectifying potassium channel (GirK), and voltage-gated K channel (KCNQ), have been recently linked to Aβ pathophysiology in the hippocampus. Specifically, by using Aβ25-35, we previously found that GirK conductance is greatly decreased in the hippocampus, and similar effects have also been reported on KCNQ conductance. Thus, in the present study, our goal was to determine the effect of A*β* on the transcriptional expression pattern of 17 genes encoding neurotransmitter receptors and associated channels which maintain excitatory-inhibitory neurotransmission balance in hippocampal circuits, with special focus in potassium channels. For this purpose, we designed a systematic and reliable procedure to analyze mRNA expression by reverse transcriptase quantitative polymerase chain reaction (RT-qPCR) in hippocampal rat slices incubated with A*β*_25-35_. We found that: 1) A*β* down-regulated mRNA expression of ionotropic GluN1 and metabotropic mGlu1 glutamate receptor subunits as previously reported in other AD models; 2) A*β* also reduced gene expression levels of GirK2, 3, and 4 subunits, and KCNQ2 and 3 subunits, but did not change expression levels of its associated GABA_B_ and M1 receptors, respectively. Our results provide evidence that A*β* can modulate the expression of these channels which could affect the hippocampal activity balance underlying learning and memory processes impaired in AD.

## Introduction

Lastest findings support the emerging concept that the effects of Amyloid-*β* (A*β*) in Alzheimer disease (AD) initially center on subtle alteration of synaptic function and, thus, precede synapse loss, plaque accumulation, the formation of tangles and neurodegeneration [1]. In this scenario, A*β* would affect neuronal activity at the molecular, synaptic or network level acting on a particular receptor/channel and inducing an imbalance between excitatory and inhibitory neurotransmission systems in relevant areas for learning and memory processes that might underlie the synaptic dysfunction found before the neurological AD deficits [2–4].

In AD, the hippocampus is one of the brain regions firstly affected. This region is a highly organized structure playing a pivotal role in learning and memory processes by the accurate balance between excitatory and inhibitory systems [5]. Memory deficits and disorientation are the first symptoms of AD [6,7] which brought up the hypothesis that hippocampal synaptic transmission and plasticity may be impaired in experimental models of the disease [2,8–11]. Humans with AD, particularly early-onset AD and familial AD, have an increased incidence of convulsive seizures [12]. Furthermore, AD transgenic models, as human amyloid precursor protein (hAPP) transgenic mice, present epileptiform activity and the downstream consequences of such aberrant hippocampal network synchronization and cognitive impairments [13]. Therefore, synaptic depression and aberrant network activity coexist in AD and seem to be mechanistically related [1,3]. It has also been reported that restoring hippocampal inhibitory cells activity and inhibitory synaptic currents is able to reduce premature mortality, network hypersynchrony, and memory deficits in hAPP mice [14]. Thus, experimental manipulations that prevent network hyperexcitability would provide key insights into the pathogenesis of AD and open new therapeutic approaches.

In this regard, several mechanisms of *loss-of-function* of sodium [14] or potassium [15–17] channels (Kv4.2, KCNQ and GirK channels), which control neuronal excitability, have been recently proposed to contribute to the alteration in AD hippocampal inhibitory neurotransmission, and to the subsequent network hyperactivity and hypersynchrony [18]. In particular, we have recently showed for the first time that A*β*_*25–35*_ decreases GABA_B_ currents in CA3 pyramidal neurons, which likely occurs by decreasing GirK channels conductance, and leads to hippocampal hiperexcitability *in vitro* [4,16]. However, the molecular mechanisms that could underlie the above A*β*-induced functional changes, i.e, synaptic dysfunction and hippocampal neurotransmission imbalance, remain mostly unrevealed, particularly at the transcriptional level [2,4]

Therefore, we have studied the transcriptional expression of neurotransmitter receptors and associated channel genes involved in neuronal excitability control, that may more likely be targeted by A*β* [4]. We developed and validated a highly sensitive reverse transcription-qPCR (RT-qPCR) assay to quantify relative mRNA levels in rat hippocampal slices incubated for long periods (up to two hours) with A*β*_*25–35*_ (the same preparation used in our previous electrophysiological studies [16]). We standardized RNA quality control to check RNA integrity maintenance in hippocampal slices and also calculated unbiased stability values of putative valid reference genes to achieve accurate and reproducible mRNA quantification in the hippocampal slice preparation. Specifically, we analyzed the modulation by A*β* of mRNA levels of 17 receptors/channels genes that participate in excitatory and inhibitory neurotransmission involving glutamatergic, GABAergic and muscarinic systems in the rat hippocampus [4]. For the study of the glutamatergic system, NMDA, AMPA and Group I of metabotropic receptors were selected while for the cholinergic system, M1 receptor and subunits of its effector, KCNQ channel were analyzed. Finally, in the GABAergic system, subunits of GABA_B_ receptor and its effector, GIRK channel, were studied [4]. Since it has been reported that A*β* early soluble forms anomalously increase activity-regulated cytoskeleton-associated protein (Arc) levels in AD patients and animal models [19], we also analyzed its mRNA expression as a positive control to validate our results.

In summary, our data indicate that A*β* modifies the mRNA expression levels of *GluN1* and *mGlu1* glutamatergic subunits as expected by previous reports [20,21], but also decreases the mRNA expression levels of different subunits of two potassium channels, *GirK2-4* and *Kcnq2-3*, that control neuronal excitability and have been recently linked to hippocampal AD pathophysiology. We suggest that these impairments at the transcriptional level could contribute to the final excitatory-inhibitory hippocampal neurotransmission imbalance that causes aberrant network activity and early cognitive impairment found in AD models [1,3,4].

## Methods

### Animals

Experiments were carried out on Wistar rats (50–100 g; P23-33) obtained from an authorized distributor (Criffa Laboratories, France). All animal procedures were reviewed and approved by the Ethical Committee for Use of Laboratory Animals of the University of Castilla-La Mancha, and followed the European Communities Council guidelines (86/609/EEC).

### Preparation of slices

**Fig 1:**
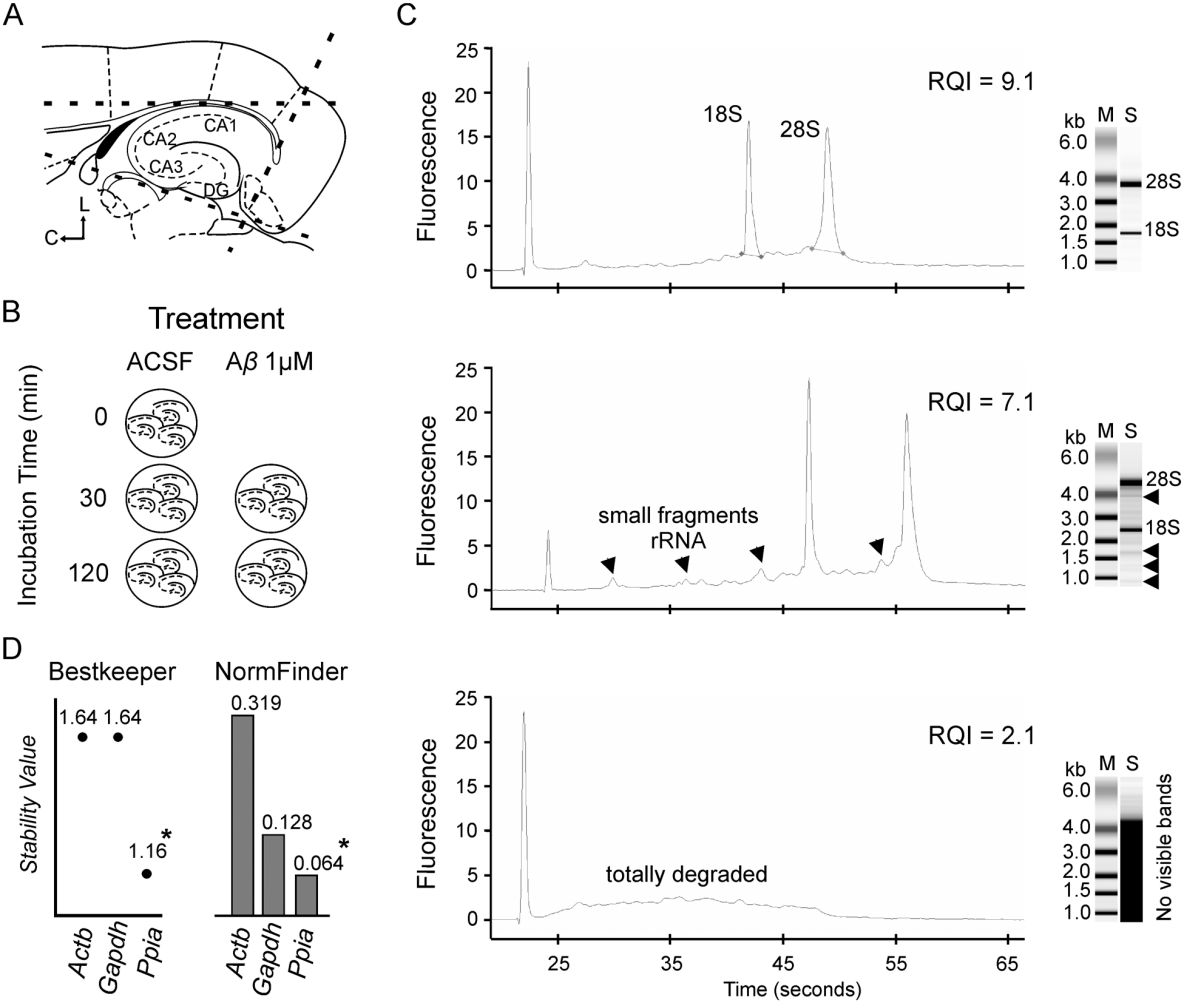
Experimental design, RNA integrity category examples and reference gene selection. **(A)** Horizontal hippocampal brain slices from one hemisphere were obtained using a dissecting microscope. Thick dashed lines indicate the cutting delimitation area. C, Caudal; L, Lateral. **(B)** Schematic representation of hippocampal slices incubation procedure. Slices were incubated in ACSF or A*β* 1°μM during 30 or 120 min. At time zero, hippocampal slices were considered as raw tissue and mRNA level for these samples 100% since incubation effects had no begun. All experiments were performed at room temperature and bubbled with carbogen gas. **(C)** Representative examples of electropherogram of different RNA samples revealing RNA integrity levels and virtual images of the gels for each sample. Left column (M), molecular weight marker; Right column (S), RNA sample. RQI values are ranged from 10 (intact) to 1 (totally degraded). The gradual degradation of RNA extracted from hippocampal slices was reflected by a continuous shift towards shorter fragment sizes. The first peak found in all traces corresponds to a molecular weight marker. Electropherogram plots from top to bottom: (Top) Profile of RNA with RQI = 9.1. The peaks correspond to 18S and 28S ribosomal subunits. In this case, there are no small peaks in profile which would indicate RNA degradation. Two bands corresponding to the 28S and 18S ribosomal RNA respectively. The greater thickness of the band corresponding to 28S indicates higher concentration compared to 18S subunit. (Middle) Profile of RNA with RQI = 7.1. Arrows indicate the different peaks from degraded RNA fragments which appear at different time points along the reaction. In the sample are arrowed degraded fragments of different sizes. (Bottom) RNA profile with RQI = 2.1. Plot corresponding to highly degraded RNA can be observed. No bands can be distinguished. **(D)** The BestKeeper and NormFinder softwares were used to calculate the most stable gene among the 3 reference genes, in ACSF and A*β* incubation. For both softwares the most stable gene is that with the lowest stability value. Asterisk indicates the selected gene (Ppia) as the most stably expressed reference gene. *Actb*, β-actin; *Gapdh*, Glyceraldehyde 3-phosphate dehydrogenase, *Ppia*, Peptidylprolyl isomerase A.

Animals were deeply anesthetized with halothane gas and decapitated. The brain was excised and rapidly immersed in modified oxygenated ice-cold (4–6°C) artificial cerebrospinal fluid (ACSF) containing: (in mM) 234 Sucrose, 4.7 KCl, 2.5 CaCl_2_, 1.2 MgCl_2_, 25 NaHCO_3_, 1.2 NaH_2_PO_4_, and 11 glucose. Horizontal hippocampal slices (350 μm-thick) were cut in cold oxygenated modified ACSF solution using a vibratome (Microm 7000smz-2, Campden Instruments Ltd, UK). Hippocampal slices from each single hemisphere were obtained using a dissecting microscope (Fig 1A), placed in an incubation chamber with ACSF containing NaCl (117 mM) replacing sucrose and maintained in a carbogen (95%CO_2_/5%O_2_) saturated atmosphere at room temperature through the incubation time, as we previously described [16,22,23].

### Preparation of A*β*_25–35_ peptide solutions

It has been proposed that A*β*_25–35_ constitutes the biologically active fragment of A*β* [24], and has been shown to induce major neuropathological signs related to early stages of AD in rats [25]. In addition, A*β*_25–35_ is reported to be more soluble and presents toxic effects more rapidly than the parent peptide A*β*_1–42_ [26] and has widely been used as a very useful tool to explore acutely the pathophysiological events related with neuronal dysfunction induced by soluble A*β* forms [16,22,27–29]. In addition, the main advantage for present work is that A*β*_25–35_ does not form ion-permeable pores in neuronal membrane but acts mainly on neurotransmission [16,27,30,31].

Therefore, A*β*_25–35_ peptide was prepared as previously described by our laboratory [16,22,29]. Briefly, the peptide was dissolved to 1 mM in bidistilled water and stored in aliquots at -20°C. Then aliquots were diluted in ACSF to required concentration of 1 μM and incubated for 24 hours at 37°C before experiments were performed [16]. Final concentration of 1 μM was chosen based on our previous functional results obtained with the same concentration in 350 μm-thick hippocampal, amygdalar and septal slices [16,22,29].

### Experimental design

Hippocampal slices were incubated at room temperature in two different conditions, 1) ACSF or 2) ACSF enriched with A*β*_25–35_ (Sigma, Poole, UK), during different time periods (0, 30, 120 minutes) (Fig 1B). Time points for gene expression analysis where chosen based on our previous *in vitro* results obtained for A*β*_25–35_ in the same experimental preparation [16]. Our previous data showed A*β*_25–35_ synaptic activity disruption as early as 20 min after perfusion of the peptide that lasted up to 2 h. In addition, maximum activity-dependent gene expression changes have been reported to take place within the time window of 0.5-2h [32,33]. Therefore 30 and 120 min were selected in order to correlate synaptic dysfunction induced by A*β* with changes in mRNA expression levels.

Finally, the slices were collected after incubation and frozen at −80°C at each time point until further processing.

### RNA extraction, RNA quality and integrity, and reverse transcription of mRNA

Total RNA was extracted from single homogenized hippocampal slices using Trizol (Invitrogen, Paisley, UK) and total RNA was purified using Rneasy Mini Kit (Qiagen, Crawley, UK). RNA samples were treated with DNase I (Qiagen, Crawley, UK) according to the manufacturer’s protocol. For all samples, RNA quantification was routinely assessed on a Nanodrop 2000c spectrophotometer (Thermo Scientific, Wilmington, USA). The quality and integrity of total RNA was analyzed by using the *RNA Quality Indicator number* (RQI), which is calculated from electropherograms obtained from an automated electrophoresis system (Experion System, Bio-Rad, USA). The electropherogram shows the RNA profile of ribosomal RNA degradation [34], according to the premise that over time RNA would accumulate as small degraded RNA with low molecular weight (Fig 1C). The RQI allowed us to classify the quality of the hippocampal samples from 1–10 (Table 1). The criterion for selection of the RNA samples used for subsequent cDNA synthesis and gene expression analysis was RQI ≥ 7.5 [34].

**Table 1:** RNA integrity and quality. RNA quality indicator (RQI) for hippocampal slices and percentage of samples for each RQI value range.

RQI	%	RNA Quality
RQI ≤ 6	2.5	Very degraded
6< RQI ≤ 7.5	15	Lightly degraded
7.5< RQI ≤ 8.5	32.5	Good quality
8.5 < RQI ≤ 10	50	High quality

cDNA synthesis was performed by reverse transcription (RT). 0.198 μg of total RNA for each sample were reversed transcribed using random nonamers 2 μM (Sigma, Poole, UK), oligo(dT)_20_ primers 1 μM (Promega, Wisconsin, US), dNTPmix 0.5 μM, (Promega, Wisconsin, US) in a *RT mix* volume of 13 μl per sample. Then we performed a “two step” amplification cycle: 1) *RT mix* was incubated at 65°C for 5 min in a T-Professional Thermocycler (Biometra, Gottingeng, Germany) for primers annealing and 2) RT was performed adding to RT mix Buffer First-Strand 5X (Invitrogen, Paisley, UK), DTT 0.1 M ribonuclease Inhibitor 40 unit/ μl (Promega, Wisconsin, US) and Superscript TM III RT (Invitrogen, Paisley, UK) in a final volume of 20 μl per sample. Then, RT mix was incubated at 25°C for 5 min, 50°C for 50 min and 70°C for 15 min.

### Quantitative polymerase chain reaction (qPCR)

qPCR reactions were performed with equal amount of cDNA template, TaqMan fast Universal Master Mix (2x) without AmpErase UNG and TaqMan Gene Expression Assays (20X) (Applied Biosystems, Carlsbad, US) for the 18 genes of interest (see S1 Table) according to the manufacturer’s protocol. The following thermal cycling specifications were performed on the ABI 7900 Fast Real-Time PCR system (Applied Biosystems, Carlsbad, US): one cycle at 95°C for 20 s, 40 cycles at 95°C for 3 s and one cycle 30 s at 60°C. Unless otherwise stated, all qPCR reactions were performed with 5 biological samples and each sample had a technical duplicate. Negative control RT samples (Non RT; samples without transcriptase for detection of genomic DNA contamination) and Non template controls, NTC-RT; samples that contained only RNasa/DNase free water, for detection of primers dimmers and contamination) were always included, as well as samples without cDNA template, NTC-qPCR (contained only RNasa/DNase free water for detection of probe’s degradation in qPCR reaction). No product was synthesized in negative controls, indicating that RT and qPCRs reagents were not contaminated. qPCR experiments were performed in accordance with MIQE (Minimum Information for Publication of Quantitative Real-Time PCR Experiments) guidelines [35].

For relative mRNA quantification C_q_ values (C_q_, standard name for threshold cycle or crossing point value according to the Real-Time PCR Data Markup Language, RDML guidelines, [36]) were calculated in each sample set for reference and target genes. The expression of each target gene relative to a normalizing factor (a reference gene that was chosen accordingly to its stability; see section below) was calculated using the 2^−ΔCq^ method [37,38] as described by the manufacturer (Applied Biosystems: User Bulletin 2). Changes in gene expression through the time course were reported as percent changes relative to basal control expression at time zero (t = 0 min, i.e. before A*β* was added to the incubation media).

### Reference gene selection

In order to compare the relative expression levels of A*β*-targeted genes following a standardized and reliable procedure, data were normalized based on the most stable reference gene in our experimental design.

As selection of suitable reference genes is critical for data normalization and interpretation, two steps were performed to choose the most stable reference gen [39]. First, the stability of three putative commonly used reference genes [40], *Actb* (β-actin), *Gapdh* (Glyceraldehyde 3-phosphate dehydrogenase), and *Ppia* (Peptidylprolyl isomerase A) was analyzed. Relative mRNA levels of reference genes were studied for all experimental conditions. C_q_ value for each reference gene was normalized to C_q_ mean value for t = 0, and mRNA level for these samples was considered 100%. The analysis revealed that mRNA levels of the three candidate reference genes were steady as expression levels of the three genes were no significantly affected by time incubation *(Actb*: F_(2,19)_ = 0.387, *p* = 0.684; *Gapdh*: F_(2,19)_ = 0.643, *p* = 0.537; *Ppia*: F_(2,18)_ = 1.921, *p* = 0.175), nor by A*β* incubation (*Actb*: F_(1,19)_ = 0.432, *p* = 0.519; *Gapdh*: F_(1,19)_ = 0.157, *p* = 0.696; *Ppia*: F_(1,19)_ = 2.928, *p* = 0.104), suggesting that any of them would be appropriate as reference gene for our experimental conditions.

Second, to determine the most stably expressed reference gene among the different candidates, the raw C_q_ values obtained for each sample for the candidate endogenous control genes were processed by two different algorithms: NormFinder v0.953 [41] and BestKeeper v1 [42] applications. These bioinformatics packages calculate a stability value, whereas a lower value means a higher stability in gene expression. The genes were ranked according to these obtained gene stability values and that with the lowest value chosen for data normalization.

Using this two-step approach that includes analysis by bioinformatics algorithms of expression stability of plausible reference genes allowed us to select the most stable gene based on an objective value and exemplify the importance of selecting a particular reference gene among different candidates to properly standardize the analysis and quantify relative mRNA levels.

### Statistical analysis

Results were expressed as mean ± standard error of the mean (SEM). All calculations were performed using SPSS version 20 software (SPSS Inc., Chicago, IL)., Comparisons of mRNA relative expression or of RNA integrity were made using two way ANOVA and followed by Bonferroni *poshoc* test when the distribution of the variables was normal. If the Levene’s test for normal distribution was significant then data were normalized by logarithmic (log) transformation. When the variables did not show normal distribution after transformation, comparisons were made using non-parametric tests. Significance level was set at *p*≤0.05.

## Results

### Standardization of the analysis of mRNA expression patterns in hippocampal slices

In order to standardize our RT-qPCR study, unbiased values were calculated to determine both: i) integrity and quality of the RNA extracted from hippocampal slices and ii) stability of reference genes in our experimental design.

### RNA integrity and quality

Total RNA was extracted and purified from hippocampal slices (n = 44). RNA integrity was analyzed for all samples using RQI value, which was calculated based on the electropherogram areas that corresponded to the regions of 28S, 18S and pre-18S ribosomal RNA (Fig 1C). Hippocampal total RNA quality was also checked by virtual gel images (Fig 1C, right column). As shown in Table 1, RQI values were divided into four groups according to our pre-established criterion. Data showed that more than 80% of the samples presented total RNA of highest quality (Table 1), confirming that the model would be appropriated to study the effects induced by A*β* on the RNA obtained from the slice. No significant differences in RNA integrity were found neither during A*β* incubation (F_(1,35)_ = 0.206; *p* = 0.652) nor time (F_(2,35)_ = 0.826; *p* = 0.446).

### Selection of the most stable reference gene

As indicated in the methods section, stability of the possible reference genes (*Ppia*, *Gapdh* y *Actb*) in our experimental conditions was first evaluated. Among suitable candidates confirmed by this first analysis, the most stably expressed reference gene (i.e. the one with lowest variation and highest stability across biological samples) was finally selected for analysis of relative expression levels of target genes after A*β* treatment in hippocampal slices.

mRNA expression levels of the three proposed genes (*Ppia*, *Gapdh* y *Actb*) were found to be steady (see methods section) so they were all considered as suitable candidates for later bioinformatic analysis of stability. To select the most stable reference gene across the tested ACSF and A*β* conditions, the expression stabilities of the 3 candidates, *Ppia*, *Gapdh* y *Actb*, were analyzed with two different software tools, NormFinder Program [41] and BestKeeper Program [42]. After data were analyzed and genes ranked by both programs, the lowest rank represented the most stably expressed reference gene, whereas the highest rank represented the least stably expressed reference gene. Across the three candidates, *Ppia* was identified as the most stably expressed reference gene by both programs (Fig 1D) and used for further analysis.

### A*β* effects on hippocampal gene expression

**Fig 2:**
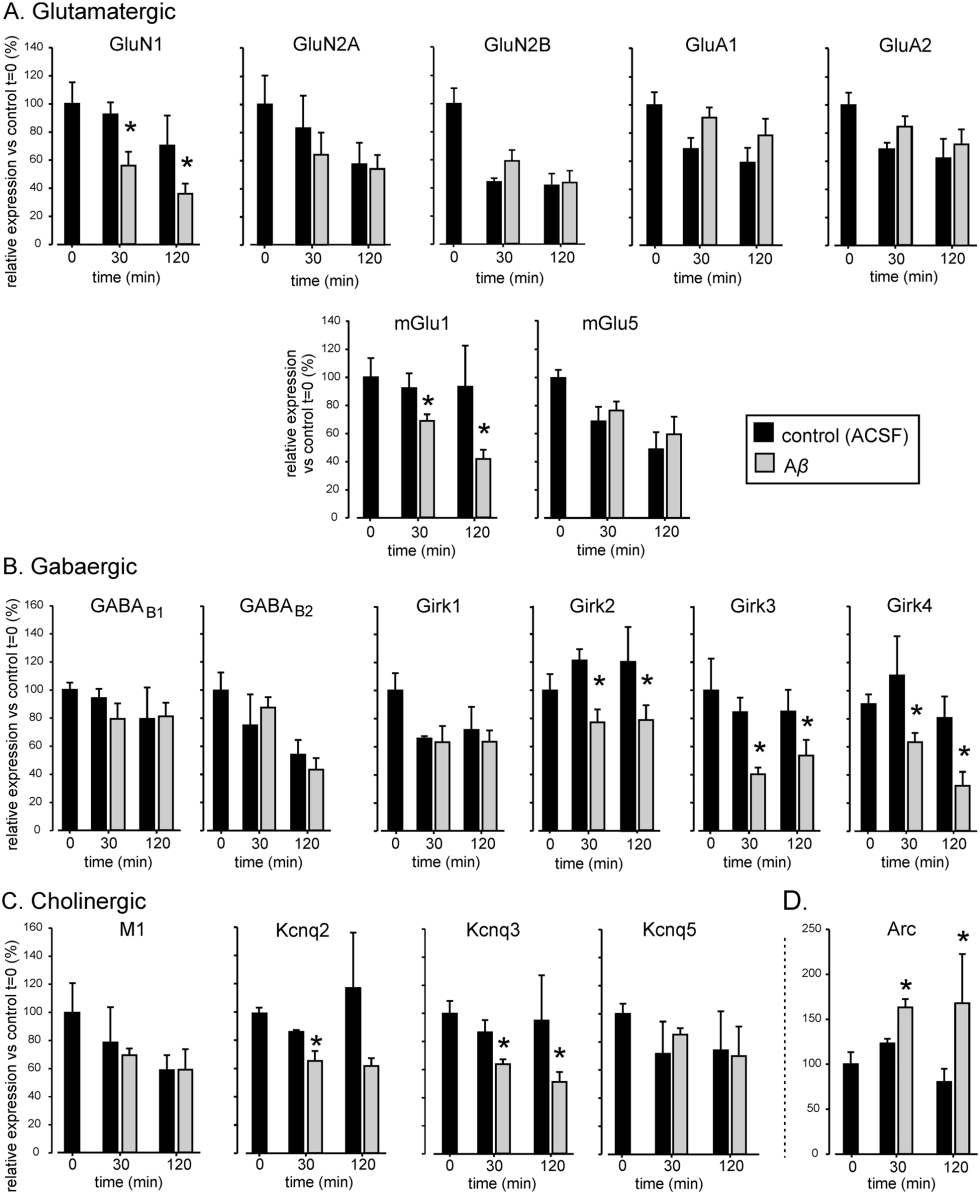
Relative expression of the main receptor and channel genes involved in excitatory and inhibitory hippocampal neurotransmission systems during A*β* incubation. Expression levels of mRNA for **(A)** glutamatergic **(B**) cholinergic and **(C)** GABAergic genes, at 0, 30 and 120 min after ACSF (control) or A*β* incubation, were analyzed by qPCR and normalized to *Ppia*, the most stably expressed reference gene in our study. Normalized 2^–ΔCq^ expression for each gene and time point is presented as percentage of gene expression at time 0 (t = 0 min) which is considered as 100% basal control expression. **(A)**. Relative mRNA expression levels of glutamatergic NMDA receptor subunits *GluN1*, *GluN2A* and *GluN2B*, AMPA receptor subunits *GluA1* and *GluA2*, and metabotropic receptor *mGlu1* and *mGlu5*. **(B)** Relative mRNA expression levels of GABA_B_ receptor subunits *GABA*_*B1*_ and *GABA*_*B2*_, and GABA_B_ receptor effector GIRK channel subunits *Girk1*, *Girk2*, *Girk3* and *Girk4*. **(C)** Relative mRNA expression levels of cholinergic M1 receptor and M1 receptor effector KCNQ channel subunits *Kcnq2*, *Kcnq3* and *Kcnq5*. **(D)** Relative mRNA expression levels of activity-regulated cytoskeleton-associated protein, *Arc*, at 0, 30 and 120 min after ACSF or A*β* incubation are also shown. Note that y-scale in D is different to A-C. Data are presented as mean ± standard error of the mean (SEM). *p < 0.05. A*β*, amyloid-beta; *Ppia*, Peptidylprolyl isomerase A; *ACSF*, artificial cerebrospinal fluid.

In order to achieve the main aim of the present study, i.e to investigate the effect of A*β* on hippocampal expression of genes related to glutamatergic, cholinergic and/or GABAergic neurotransmission, we studied the expression pattern of 17 subunits of receptors/channels than control neuronal excitability, using *Ppia* as reference gene. As stated above, mRNA expression of *Arc* was analyzed as positive control. Soluble A*β* has been shown to induce Arc expression itself [19,43] and *Arc* overexpression has been linked to dysfunctional learning, suggesting a molecular basis for the specific loss of memory function in early AD. Accordingly, our results showed A*β* to significantly increase *Arc* mRNA levels at 30 and 120 minutes incubation times (Fig 2D) (F_(1,9)_ = 5.428, *p* = 0.045) suggesting a role in neurophysiological alterations of our AD experimental model.

mRNA levels for ionotropic NMDA and AMPA receptor subunits were found unchanged except for NMDA *GluN1* subunit, which was significantly decreased by A*β* (Fig 2A) after 30 min and 2 h incubation (F_(1,9)_ = 7.242, *p* = 0.025).

Regarding metabotropic mGlu glutamate receptors, in our experimental conditions, A*β* induced *mGlu1* mRNA levels to decrease (30 min, *p* = 0.037; 120 min, *p* = 0.034) while *mGlu5* levels were not significantly modified (Fig 2A).

A*β* effects on mRNA expression of GABA_B_ receptors and GIRK channels, second messenger effectors of GABA_B_-mediated neurotransmission, were also analyzed. mRNA levels of GABA_B_ receptor subunits, *GABA*_*B1*_ and *GABA*_*B2*_ were not modulated by A*β*. However, the subunits of its effector, GirK channel, were altered by A*β* incubation (Fig 2B). Thus, mRNA expression levels for *GirK2*, *GirK3*, and *GirK4* were found to be reduced at 30 and 120 minutes of A*β* incubation when compared to ACSF treated samples (*GirK2*: F_(1,9)_ = 9.187, *p* = 0.014; *GirK3*: F_(1,8)_ = 12.293, *p* = 0.008; *GirK4*: F_(1,11)_ = 9.605, *p* = 0.010).

Relative expression of genes related to the cholinergic system were also analyzed and 30-minutes after A*β* incubation mRNA levels of muscarinic receptor subtype M1, *Chmr1*, did not changed. mRNA levels of KCNQ channel subunits (that ensemble to form M1 receptor effector) were more vulnerable and exhibited a down-regulation (Fig 2C; *Kcnq2*, *p* = 0.035; *Kcnq3*, *p* = 0.037). In addition, after 120-minutes A*β* incubation only induced a significant decrease of *Kcnq3* subunit expression (Fig 2C, *p* = 0.034). Levels of *Kcnq5* were not altered by A*β*.

## Discussion

In the present work we studied the modulation by A*β*_*25–35*_ of gene expression of neurotransmitter receptors and associated channels which maintain excitatory-inhibitory balance in hippocampal circuits. We provided evidence of two targets down-regulated by A*β*, KCNQ [15,27] and GirK [16] channels, that participate in controlling neuronal excitability and have been recently related with AD pathogenesis by our laboratory and others. We also showed that A*β* reduced gene expression of glutamate ionotropic GluN1 and metabotropic mGlu1 receptor subunits. Both potassium conductances have previously been shown to be decreased by A*β*, which could contribute to the hyperexcitability and network dysfunction that underlie early stages of AD [1,2].

In the present study we used the hippocampal slice preparation that was incubated with A*β* up to 2h at room temperature. Therefore, as a crucial prerequisite for reliable qPCR conclusions we designed and standardized an unbiased method to minimize the influence of sample manipulation, time incubation and temperature in RNA integrity and data normalization in hippocampal slices. RQI number for each sample was considered and unbiased stability values for reference genes were calculated to ensure relevance, accuracy, correct interpretation, and repeatability of the assays. Therefore, our present experimental design provides reliable and consistent data concerning A*β*-modulation of key receptor/channel genes involved in hippocampal network excitability control, and lead us to consider it as a valid model for studying the acute effects induced by A*β* on gene expression profile of hippocampal rat slices.

### Quality and Integrity of RNA Samples (RQI)

Only samples with RQI ≥ 7.5 were included in our study, i.e. good or high quality RNA. Our data showed that neither time nor A*β* incubation affected RNA integrity of hippocampal slices. Classically, RNA integrity has been evaluated using agarose gel electrophoresis, which allows studying the ratio of two bands comprising the 28S and 18S ribosomal RNA species. However this approach has been shown to be inconsistent, since relies on human interpretation of gel images [34]. In the present work, the RQI number, an algorithm based in the electropherogram areas corresponding to the regions 28S, 18S and pre-18S, was used both to evaluate RNA integrity in an unambiguous way [34] and as an objective criterion to evaluate the quality of the samples [44].

### Reference genes

BestKeeper and NormFinder algorithms are novel approaches for examining potential reference genes to select the most stable housekeeping gen for a given set of conditions. Following the MIQE recommendations, in the present work 3 putative reference genes, *Actb*, *Gapdh* and *Ppia*, were investigated [40,45] and ranked according to their expression stability by both algorithms. In our experimental conditions, mRNA levels for the three genes were maintained, so from a *classical* point of view, they all could be considered suitable to be used as reference genes [46]. However, *Ppia* had been previously shown to be more stable than *Gadph* or *Actb* in different experimental preparations [39,47]. In agreement, in our study *Ppia* was selected for data normalization among the three candidate genes as both stability BestKeeper and NormFinder stability algorithms found *Ppia* as the most stable expressed reference gene in our experimental conditions. This result further contributes to objectify and standardize a procedure for relative gene expression analysis in hippocampal slices, and indicates that searching for appropriate reference gene or genes in any experimental design is a crucial step. Furthermore, we have shown for the first time a method to analyze the integrity of total RNA in hippocampal slices which have been used for other purposes such as electrophysiology [9]. Thus, this approach represents a powerful tool to further analyze the molecular mechanisms involved in hippocampus networking.

### A*β* target genes in the hippocampus

Considering the large body of information available about its cytoarchitecture and synaptic organization, the hippocampal slice preparation is an election model suitable to be used in AD neuropharmacological studies [9]. The main advantage comes from the possibility of being used in highly controlled *in vitro* environments, thus preserving the cellular interactions of the brain *in vivo*, which allows analysis of drug effects, at the cellular and subcellular levels, on different excitatory and inhibitory neurotransmission systems [48–50].

Recent works suggest that A*β* impact transcription in several important neuronal pathways preceding neurodegeneration [51]. These impairments might underlie early A*β*-induced synaptic dysfunction and the imbalance observed in hippocampal networks which produce aberrant excitatory neuronal activity and contribute to cognitive deficits in AD models [1,8,52]. Although the precise mechanisms remain unknown, the synaptic impairments that lead to neural network hyperactivity, an early event in AD [18], is likely the mechanism involved. Along the different A*β* targets that could participate in controlling the excitability of the hippocampal networks, two potassium channels have been very recently identified: voltage-gated K channel, Kv7 (also termed KCNQ) [15] and G protein-coupled activated inwardly-rectifying potassium channels, GirK [16]. In the present work, we found A*β* to down-regulate mRNA levels of different subunits of both type of potassium channels. We also found A*β* to reduce gene expression of glutamate ionotropic GluN1 and metabotropic mGlu1 receptor subunits.

#### Glutamate receptors and A*β*

Despite the wide evidence that A*β* affects glutamatergic neurotransmission, in our experimental conditions significant changes were only found in mRNA levels of NMDA and mGlu receptor subunits.

Glutamatergic neurotransmission through AMPA receptors has widely been involved in AD pathology with different results. It has been shown that A*β* oligomers reduce phosphorylation of GluA1 subunit leading to a decrease of surface AMPA receptors without evident alteration in mRNA transcripts [53]. Our results would be in accordance with these findings since we did not observed changes in mRNA expression levels. On the other hand, GluA2 subunit of glutamate AMPA receptors has been linked to A*β* neurotoxic mechanisms [54]. In synaptoneurosomes from prefrontal cortex, an increase in mRNA encoding GluA2 subunit has been reported to be paralleled by elevated expression of the corresponding protein in incipient AD patients [55]. However, in the latter study only one reference gene was used for data validation what might explain that we could not find such increase in our experimental conditions.

NMDA receptors activation has been extensively involved in AD-related synaptic dysfunction [56,57]. In the present study we did not find significant changes in the mRNA expression of NMDA subunits *GluN2A* and *GluN2B* while *GluN1* was decreased by A*β*.

In accordance with our results, *GluN1* mRNA levels were also found significantly decreased in human AD cerebral cortex [21] as well as in rat brain tissue [58]. Moreover, a study evaluating alterations in the transcriptional expression of glutamatergic receptors in late stages of sporadic AD, has shown *GluN1* to be down-regulated in the hippocampus of AD patients by using qPCR and array hybridization methods [59]. It would be very interesting to know what happens in early stages of AD, when compensatory mechanisms would not have taken place yet.

Levels of *GluN2B* and *GluN2A* mRNA were decreased in susceptible regions of postmortem human AD brains, such as the hippocampus and the cortex [60]. Conversely, as in our study in rat hippocampal tissue, there is evidence that mRNA levels of and *GluN2A* and *GluN2B* subunits [61] are unchanged in AD patient's brains. It has also been reported that A*β* regulates Fyn kinase, which has been proposed to disrupt hippocampal network activity [28]. Fyn signaling drives phosphorylation of GluN2B subunit of NMDA receptor which in turn produces altered surface expression of the receptor, dysregulation of receptor function, excitoxicity and dendritic spine retraction [62,63]. The latter alterations are produced mainly at protein level, so they might not be detected at mRNA level. Further analysis is needed in this line.

Group I of metabotropic glutamate receptors (mGlu), mGlu1 and mGlu5, participates in the regulation of synaptic plasticity and postsynaptic glutamatergic excitability [64]. We found A*β* to decrease mRNA levels of *mGlu1* but no significant differences were detected in *mGlu5* mRNA expression. Similar results have been reported at the protein level in the cerebral cortex of patients with AD [20], where *mGlu1* activity is down-regulated (with no changes in *mGlu5*) by significant decrease in its expression levels, which correlated with severity of the disease. Very recently a main role of mGluR5 as a triggering factor for the pathogenesis of AD has been proposed, mainly through the interaction of Aβ oligomers with the receptor at the protein level [65]. Further studies at the mRNA level for these receptors in hippocampal neurons are still needed.

#### Potassium channels and A*β*

Our previous studies have shown that A*β* peptide perfusion of hippocampal slices alters the septohippocampal neurotransmission through fimbria-CA3 synapse decreasing GIRK channel conductances [16]. These effects included depolarization of CA3 pyramidal neurons, input resistance increase, and decrease of the GABA_B_-mediated inhibitory neurotransmission [16]. Thus, GIRK channels might play a role as a part of the mechanisms underlying the loss of septohippocampal rhythmic state that promote failures in synaptic plasticity and memory formation observed in AD patients [4,16]. In addition to this acute effect of A*β* on GIRK function, in the present study we found that A*β* incubation also induced down-regulation of three GIRK subunits, *GirK2*, *GirK3* and *GirK4* at the mRNA level. mRNA levels of *Girk2 and Girk3* were altered at 30 and 120 minutes. GIRK2 subunit is widely expressed in the brain, forms functional heterotetramers (GIRK1–GIRK2, GIRK2–GIRK3) and homotetramers (GIRK2–GIRK2) and has been implicated in various functions and pathologies [66,67], such as learning and memory, reward, motor coordination, and Down syndrome. Mutations in GIRK2 subunit have been previously reported to reduce LTP and increase LTD in hippocampus [66]. These effects are especially relevant in Down syndrome, where cerebral A*β* accumulation is greatly accelerated and leads to invariant early-onset AD neuropathology [68–70]. Therefore, our results at the mRNA level might likely imply a decrease of GIRK2 subunit at the protein level (once translated and trafficked to the membrane) and would be in accordance with the key role of GIRK2 subunit in GIRK channel function and control of hippocampal excitability.

We also found a marked down-regulation of *Girk4* mRNA levels through the time course of A*β* incubation. Protein expression levels of GIRK4 are very low in most regions of the CNS when compared with the abundant expression of other GIRK subunits in the rat brain [71]. In the hippocampal formation, GIRK4 expression has been shown to be more prominent in CA3 pyramidal neurons but only moderate to low in both CA1 neurons and dentate gyrus granule cells [72,73]. However, although immunohistochemical techniques have failed to show a robust staining of GIRK4 expression in hippocampal formation [74,75], *Girk4* knock-out mice has exhibited impaired performance in spatial learning and memory tests [75]. It is plausible that qPCR allowed us to find *Girk4* transcript modulation by A*β* in hippocampal slices whereas histological techniques are not able to detect these subtle changes, as occurs in other nervous tissues with other potassium channels [38]. Thus, our results show a new reliable method to detect changes in *Girk4* gene expression which may explain the impairment of hippocampal networks [75].

In summary, our data [16] suggest that A*β* could interfere with GIRK channel functioning acutely by a mechanism non described yet (vg. altering channel relationship with lipid membrane or acting directly on the channel). This acute effect could be linked to changes in the gene expression of the channel that will reinforce the acute effect (changing channel expression or turnover) and may explain long-term deficits found in transgenic mice models of AD. However, the hypothesis of two independent mechanisms (acute and at the mRNA level) cannot be ruled out.

On the other hand, our results have shown that A*β* did not significantly modulate M1 muscarinic mRNA levels. Among different muscarinic receptors, M1 subtype has been widely related with AD [76–78]. However it has been reported that A*β* has no influence on M1 muscarinic receptors or gene expression but on the receptor/G-protein interaction [79]. KCNQ channels are the effectors of muscarinic receptors. KCNQ family comprises five members, KCNQ1–5, encoded by *Kcnq1*–*5* genes [80]. In particular, it has been reported that KCNQ2–KCNQ3 heteromers [81], KCNQ4 [82], and KCNQ5 [83], participate in the maintenance of M-type K currents and play a crucial role in the regulation of neuronal excitability [84]. The ‘classical’ M-channel is composed of KCNQ2–KCNQ3 heteromers [81,84]. mRNA levels of both subunits are found to be early down-regulated by A*β* in the hippocampus in our study. Consistent with our findings regarding *Kcnq2–3* subunits alteration, it has been recently shown that decrease in M-type K currents in medial septal area neurons may be an integral part of AD pathophysiology [27], explaining why M-type K current blockers fail to improve cognition in AD clinical trials [85]. It has also been reported that *Kcnq2* gene is down-regulated by A*β* in the hippocampus [15] and, in this conditions, M-type K current reduction would rapidly increase the excitability of hippocampal principal and GABAergic interneurons, impairing rhythmicity and synchronic activities [15,27]. At 120 minutes A*β* did not induce significant differences in *Kcnq2* gene expression, suggesting some kind of compensatory response to restore the pre-A*β* incubation status as also occurs with A*β* long term effects [15,27]. Finally, it has recently been demonstrated that KCNQ5 controls excitability and function of hippocampal networks through modulation of synaptic inhibition [86]. However, in our experimental model its mRNA expression levels were found unchanged suggesting that further research is needed in this interesting direction but for now, KCNQ5 may not be linked to AD physiopathology.

## Conclusions

Based on an objective standardized and validated analysis method we show the modulation of mRNA expression by A*β*_*25–35*_ in incubated hippocampal rat slices. In addition to the reported modulation of hippocampal glutamatergic neurotransmission by A*β*, we provided evidence of two targets for A*β* action at the molecular level that participate in controlling neuronal excitability and have recently been related with AD, KCNQ [15,27] and GirK [16] channels. Both potassium conductances have been shown to be decreased by A*β*, which contributes to the hyperexcitability and network dysfunction that underlie early stages of AD [1,2]. Our data show changes in mRNA expression of KCNQ and GirK subunits suggesting one potential A*β* mechanism at the transcriptional level that could have functional consequences such as increased network excitability. The presence of seizures and epileptiform activity in AD subjects support the hypothesis that aberrant network activity contributes causally to synaptic and cognitive deficits [13] and that a reduction in excitability with enhanced rhythmicity may be a more promising therapeutic approach in AD [1,3,14,27,87,88]. Thus, antiepileptic drugs such as KCNQ openers [89] or GirK activators [66,67,90] might ameliorate those deficits although no clinical evidence is still available [4].

## Supporting Information

S1 Table Details and primers of target and reference genes. (XLSX)
